# Supplementary material for: A zero inflated log-normal model for inference of sparse microbial association networks
Source: PLoS Comput Biol. 2021 Jun 18;17(6):e1009089. doi: 10.1371/journal.pcbi.1009089 (PMC8244920; doi:10.1371/journal.pcbi.1009089)
Supplement: S1 Text — Discussion of the issue with compositionality under the log-normal model in a high dimensional setting. (PDF) [file pcbi.1009089.s001.pdf]

S1 Text. Problem with compositionality under the gaussian assumption

If we assume that $\boldsymbol{x}$ follows a Gaussian distribution $\boldsymbol{x} \sim \mathcal{N}(\mu, \Sigma)$, then $\boldsymbol{x}^{\text{clr}}$ also follows a Gaussian distribution $\mathcal{N}(F\boldsymbol{\mu}, F\Sigma F)$. However, $F\Sigma F$ does not necessarily converge to Σ when p increases, as shown by taking the Frobenius norm of the difference between Σ and $F\Sigma F$:

$$\begin{aligned}
\|\Sigma - F\Sigma F\|_{\mathcal{F}}^2 &= \left\| \frac{2}{p} J\Sigma - \frac{1}{p} J\Sigma J \right\|^2 && (1)\\
&= \sum_{ij} \left(\frac{2}{p} \sum_k \sigma_{ik} - \frac{1}{p^2} \sum_{kl} \sigma_{kl} \right)^2 && (2)\\
&= p \sum_i l_i^2 && (3)
\end{aligned}$$

with

$$l_i = \frac{2}{p} \sum_k \sigma_{ik} - \frac{1}{p^2} \sum_{kl} \sigma_{kl}$$
