# Supplementary material for: A zero inflated log-normal model for inference of sparse microbial association networks
Source: PLoS Comput Biol. 2021 Jun 18;17(6):e1009089. doi: 10.1371/journal.pcbi.1009089 (PMC8244920; doi:10.1371/journal.pcbi.1009089)
Supplement: S2 Text — Discussion of the effect of the clr transformation in the presence of an excess of biological zeros. (PDF) [file pcbi.1009089.s002.pdf]

# S2 Text. Problem with the clr transformation when there is an excess of zeros

A key property of the clr transformation is the equivalence between log-ratios of compositional and log-ratios of absolute data. For example, if we assume that $\boldsymbol{x}_i$ is scaled by $N$,

$$\boldsymbol{y}_i = N\boldsymbol{x}_i$$

then we have

$$y_{ij}^{\text{clr}} = x_{ij}^{\text{clr}}$$

If we combine now a clr transformation with pseudo counts, we obtain $y_{ij}^{\text{clr, s-e}}$ as

$$y_{ij}^{\text{clr, s-e}} = \log(y_{ij}+1) - \frac{1}{p}\sum_{k=1}^{p}\log(y_{ik}+1)$$

If $\boldsymbol{y}_i$ contains some zero values, we will have

$$\begin{aligned} y_{ij}^{\text{clr, s-e}} &= \log(y_{ij}+1) - \tfrac{1}{p}\textstyle\sum_{k=1}^{p}\log(y_{ik}+1) \\ &\approx \log(y_{ij}) - \tfrac{1}{p}\textstyle\sum_{k,y_{ik}\neq 0}\log(y_{ik}) \\ &= \log(Nx_{ij}) - \tfrac{1}{p}\textstyle\sum_{k,y_{ik}\neq 0}\log(Nx_{ik}) \\ &= \log(x_{ij}) - \tfrac{1}{p}\textstyle\sum_{k,y_{ik}\neq 0}\log(x_{ik}) + \tfrac{1}{p}\,|k, y_{ik}=0|\log(N) \\ &= x_{ij}^{\text{clr}} + \tfrac{1}{p}\,|k, y_{ik}=0|\log(N) \end{aligned} \tag{4}$$

with $|k, y_{ik}=0|$ the number of zero entries of $\boldsymbol{y}_i$. It is apparent that log transformed ratios are not scale-invariant in the presence of zero values, and that the difference increases with the number of zeros in the vector.

Thus, if the data is very sparse, and if the sequencing depth further has high variance, then the relevance of the clr transformation diminishes.
