# Supplementary material for: A zero inflated log-normal model for inference of sparse microbial association networks
Source: PLoS Comput Biol. 2021 Jun 18;17(6):e1009089. doi: 10.1371/journal.pcbi.1009089 (PMC8244920; doi:10.1371/journal.pcbi.1009089)
Supplement: S3 Text — Description of the one step EM procedure. (PDF) [file pcbi.1009089.s003.pdf]

S3 Text. One step EM

Assuming the following probabilistic model:

$$\begin{aligned} \boldsymbol{z}_i &\sim \mathcal{N}(\boldsymbol{\mu}, \Sigma) \\ \tilde{y}_{ij} &= I(z_{ij} > \delta_j) z_{ij}, \end{aligned} \tag{5}$$

an EM argument can show that the penalized likelihood can be increased by maximizing:

$$L^1(\Sigma^{-1}) = \mathbb{E}_{Z|\tilde{Y}} \left(\log(\mathbb{P}(Z|\boldsymbol{\mu}, \Sigma^{-1})) \right) - \lambda \|\Sigma^{-1}\|_1 \tag{6}$$

$$= \frac{n}{2} \log |\Sigma^{-1}| - \frac{n}{2} \mathbb{E}_{Z|\tilde{Y}} \left(tr(S\Sigma^{-1}) \right) - \lambda \|\Sigma^{-1}\|_1 + \text{const} \tag{7}$$

where $S := \frac{1}{n} \sum_i (\boldsymbol{z}_i - \hat{\boldsymbol{\mu}})(\boldsymbol{z}_i - \hat{\boldsymbol{\mu}})^T$.

When taking as initial parameters the diagonal estimates $\Sigma_0 = \text{diag}(\Sigma_{11}, \Sigma_{22}, .., \Sigma_{pp})$, the equation can be simplified with the following equality (see [1]):

$$\mathbb{E}_{Z|\tilde{Y}} \left(\text{tr}(S\Sigma^{-1}) \right) = \text{tr}(\tilde{S}\Sigma^{-1})) \tag{8}$$

where $\tilde{S}$ is the empirical covariance matrix of the posterior mean of Z, noted $\tilde{Z}$ and defined as following:

$$\tilde{z}_{ij} = \mathbb{E}_{z_{ij}|\tilde{y}_{ij}}(z_{ij})$$

We noticed here a small inaccuracy in [1], affecting equation (8) and based on the following observation (where $\boldsymbol{\mu} = 0$ for more simplicity; we also remind the reader of the notation $\Omega = \Sigma^{-1}$).

$$\mathbb{E}_{Z|\tilde{Y}} \left(\text{tr}(S\Omega) \right) = \mathbb{E}_{Z|\tilde{Y}} \left(\sum_{jk} S_{jk} \Omega_{jk} \right) \tag{9}$$

$$= \mathbb{E}_{Z|\tilde{Y}} \left(\sum_{jk} \left(\frac{1}{n} \sum_i z_{ij} z_{ik} \right) \Omega_{jk} \right) \tag{10}$$

$$= \sum_{jk} \Omega_{jk} \frac{1}{n} \sum_i \mathbb{E}_{\boldsymbol{z}_i|\tilde{\boldsymbol{y}}_i} \left(z_{ij} z_{ik} \right) \tag{11}$$

$$= \sum_{jk} \Omega_{jk} \frac{1}{n} \sum_i \mathbb{E}_{z_{ij}|\tilde{y}_{ij}}(z_{ij}) \mathbb{E}_{z_{ij}|\tilde{y}_{ij}}(z_{ik}) \tag{12}$$

$$= \text{tr}(\tilde{S}\Sigma^{-1})) \tag{13}$$

The passage from (11) to (12) relies on the equality:

$$\mathbb{E}_{\boldsymbol{z}_i|\tilde{\boldsymbol{y}}_i} \left(z_{ij} z_{ik} \right) = \mathbb{E}_{z_{ij}|\tilde{y}_{ij}}(z_{ij}) \mathbb{E}_{z_{ij}|\tilde{y}_{ij}}(z_{ik})$$

justified by the fact that Σ_0 is diagonal. This is true for all j, k when $j \neq k$ but doesn't hold when $j = k$.

References

1. Sinclair D, Hooker G. Sparse inverse covariance estimation for high-throughput microRNA sequencing data in the Poisson log-normal graphical model. Journal of Statistical Computation and Simulation. 2019;89(16):3105–3117. doi:10.1080/00949655.2019.1657116.
