# Supplementary material for: A zero inflated log-normal model for inference of sparse microbial association networks
Source: PLoS Comput Biol. 2021 Jun 18;17(6):e1009089. doi: 10.1371/journal.pcbi.1009089 (PMC8244920; doi:10.1371/journal.pcbi.1009089)
Supplement: S4 Text — Formalization of neighborhood selection as a penalized maximization problem, justifying its inclusion in the one step EM procedure. (PDF) [file pcbi.1009089.s004.pdf]

S4 Text. Neighbourhood selection as a penalized maximization problem

The argument follows unpublished work from Ambroise and Chiquet: ”solving MB is equivalent to maximize a penalized pseudo likelihood”.

The pseudo likelihood p-$\mathcal{L}$ of a gaussian vector $\boldsymbol{x}$ is an approximation of its likelihood and is defined as following:

$$\text{p-}\mathcal{L}(\boldsymbol{\mu}, \Sigma|\boldsymbol{x}) = \prod_i^p \mathbb{P}(x_i|\boldsymbol{x}_{\setminus i})$$

Let's assume that $\tilde{\boldsymbol{y}}_i$ follows a normal distribution: $\tilde{\boldsymbol{y}}_i \sim \mathcal{N}(\boldsymbol{\mu}, \Sigma)$, so we can compute the distribution of variable i conditioned on other variables:

$$\tilde{y}_{ij}|\tilde{\boldsymbol{y}}_{i\setminus j} \sim \mathcal{N}(\mu_{j|\setminus j}, \Sigma_{j|\setminus j})$$

with

$$\mu_{j|\setminus j} = \mu_j + \Sigma_{j\setminus j}\Sigma^{-1}_{\setminus j\setminus j}(\tilde{\boldsymbol{y}}_{\setminus j} - \boldsymbol{\mu}_{\setminus j})$$

and

$$\Sigma_{j|\setminus j} = \Sigma_{jj} - \Sigma_{j\setminus j}\Sigma^{-1}_{\setminus j\setminus j}\Sigma^T_{j\setminus j}$$

By taking the logarithm, we can develop

$$\log P(\tilde{y}_{ij}|\tilde{\boldsymbol{y}_{i\setminus j}}) = \frac{1}{2}\log(\frac{1}{\Sigma_{j|\setminus j}}) - \frac{1}{2\Sigma_{j|\setminus j}}(y_{ij} - \mu_{j|\setminus j})^2 + \text{const}$$

Now assume $\boldsymbol{\mu} = 0$ for simplicity, and note $\Omega = \Sigma^{-1}$. Developments for block matrix inversion give us:

$$\Omega_{j\setminus j} = -\left(\Sigma_{jj} - \Sigma_{j\setminus j}\Sigma^{-1}_{\setminus j\setminus j}\Sigma^T_{j\setminus j}\right)^{-1}\Sigma_{j\setminus j}\Sigma^{-1}_{\setminus j\setminus j}$$

and

$$\Omega_{jj} = \left(\Sigma_{jj} - \Sigma_{j\setminus j}\Sigma^{-1}_{\setminus j\setminus j}\Sigma^T_{j\setminus j}\right)^{-1}$$

We notice that:

$$\Sigma_{j\setminus j} = \frac{1}{\Omega_{jj}}$$

and

$$\mu_{j|\setminus j} = -\frac{\Omega_{j\setminus j}}{\Omega_{jj}}\tilde{\boldsymbol{y}}_{\setminus j}$$

Those terms can then be injected in the log pseudo likelihood expression:

$$\log \mathbb{P}\left(\tilde{y}_{ij}|\tilde{\boldsymbol{y}}_{i\setminus j}\right) = \frac{1}{2}\log\left(\Omega_{jj}\right) - \frac{1}{2}\Omega_{jj}\left(\tilde{y}_{ij} + \frac{\Omega_{j\setminus j}}{\Omega_{jj}}\tilde{\boldsymbol{y}}_{i\setminus j}\right)^2 + \text{const}$$

Fixing Ω_{jj} to a constant:

$$\log \mathbb{P}\left(\tilde{y}_{ij}|\tilde{\boldsymbol{y}}_{i\backslash j}\right) = -\frac{\Omega_{jj}}{2}\left(\tilde{y}_{ij} - \frac{\Omega_{j\backslash j}}{\Omega_{jj}}\tilde{y}_{i\backslash j}\right)^2 + \text{const}$$

Finally, the whole penalized log pseudo likelihood can be expressed as:

$$\begin{aligned}
\text{p-}\mathcal{L}\left(\Omega_{\text{off}}|\tilde{Y}\right) &= \sum_j \sum_i -\frac{\Omega_{jj}}{2}\left(\tilde{y}_{ij} - \frac{\Omega_{j\backslash j}}{\Omega_{jj}}\tilde{\boldsymbol{y}}_{i\backslash j}\right)^2 - \lambda\|\Omega_{\text{off}}\|_1 + \text{const} \\
&= \sum_j -\frac{\Omega_{jj}}{2}\|\tilde{Y}_{.j} - \frac{\Omega_{j\backslash j}}{\Omega_{jj}}\tilde{Y}^T_{.\backslash j}\|_2^2 - \lambda\|\Omega_{\text{off}}\|_1 + \text{const}
\end{aligned}$$

Where the parameter Ω_{off} is the off-diagonal values of Ω.

Solving the lasso regressions of the MB algorithm is therefore equivalent to maximizing the penalized pseudo likelihood with the off-diagonal values of the inverse covariance matrix as parameter.
