# Supplementary material for: A zero inflated log-normal model for inference of sparse microbial association networks
Source: PLoS Comput Biol. 2021 Jun 18;17(6):e1009089. doi: 10.1371/journal.pcbi.1009089 (PMC8244920; doi:10.1371/journal.pcbi.1009089)
Supplement: S1 Table — Assortativity coefficients (p < 10−4) of graphs for the different methods at various phylogenetic levels. (PDF) [file pcbi.1009089.s005.pdf]

S1 Table. Assortativity coefficients

Level	Spiec-Easi	Flashweave	MAGMA	ZiLN
Phylum	0.14	0.15	0.19	0.15
Class	0.09	0.11	0.13	0.12
Order	0.08	0.10	0.11	0.10

S1 Table. Assortativity coefficients
