# Supplementary figures and images for: A zero inflated log-normal model for inference of sparse microbial association networks
Source: PLoS Comput Biol. 2021 Jun 18;17(6):e1009089. doi: 10.1371/journal.pcbi.1009089 (PMC8244920; doi:10.1371/journal.pcbi.1009089)

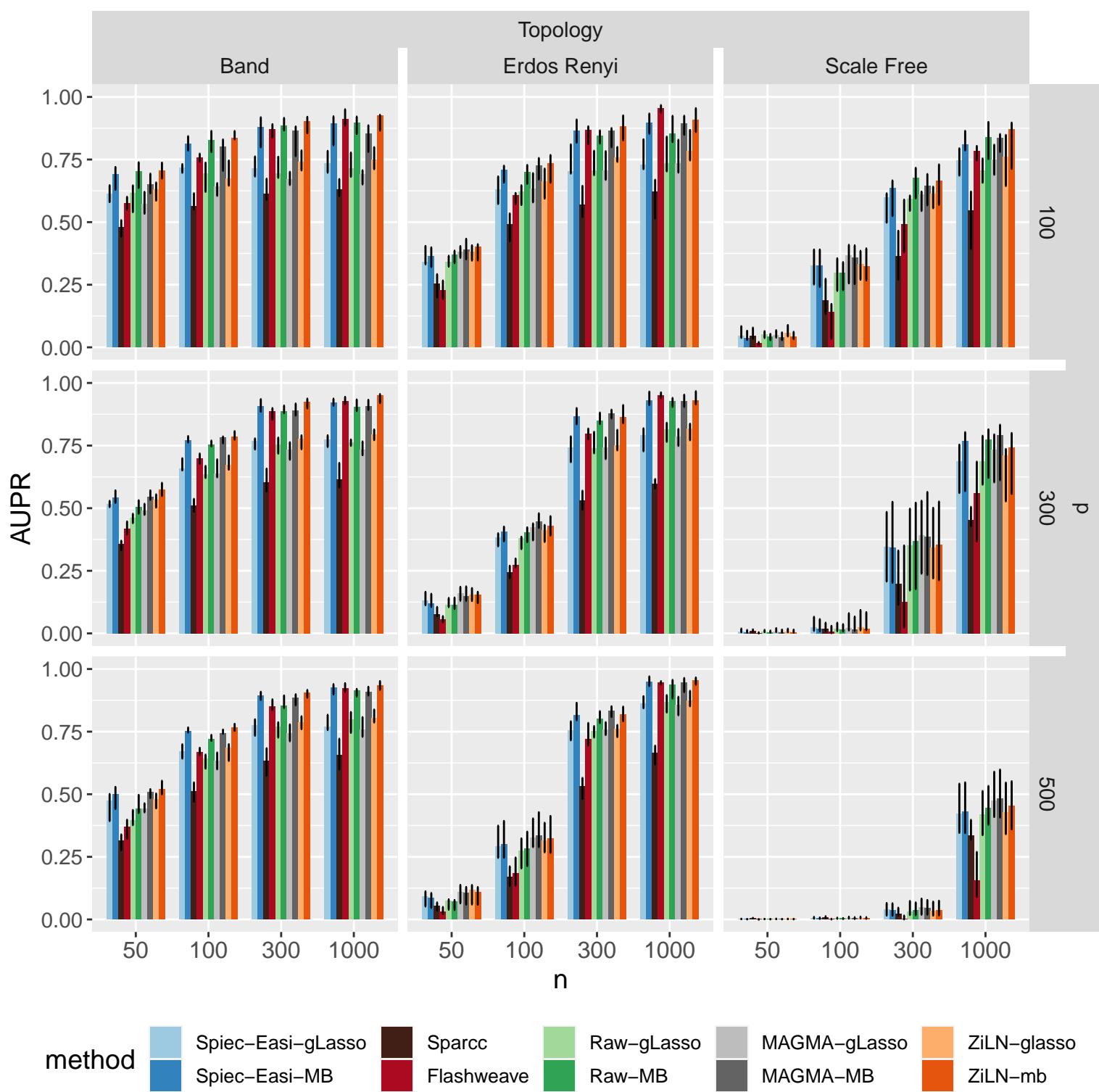

Supplement: S1 Fig — Bars represent the median over 10 runs, and error bars +25% and −25% quantiles. Each method (Spiec-Easi (blue) [23], MAGMA (gray) [24] and ours (ZiLN, orange), as well as no transformation at all (green)), was tested with two structure inference algorithms (glasso and neighborhood selection). SparCC (dark-brown) [36] and Flashweave (dark-red) [37] are two unrelated inference methods based on a distinct (orthogonal) rationale, and were included for broadening the comparisons. (PDF) [file pcbi.1009089.s006.pdf]

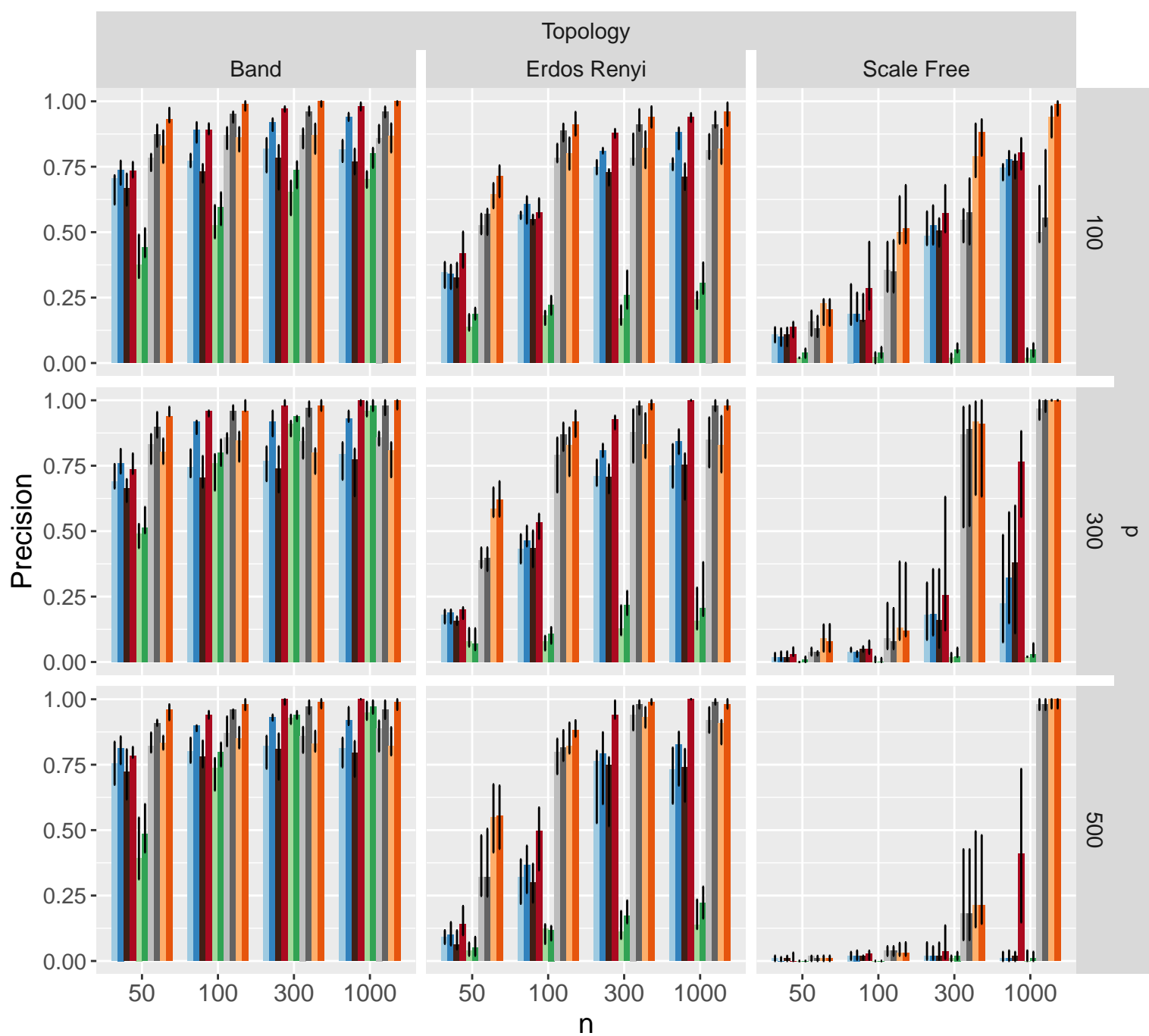

Supplement: S2 Fig — (PDF) [file pcbi.1009089.s007.pdf]
