# Supplementary material for: A zero inflated log-normal model for inference of sparse microbial association networks
Source: PLoS Comput Biol. 2021 Jun 18;17(6):e1009089. doi: 10.1371/journal.pcbi.1009089 (PMC8244920; doi:10.1371/journal.pcbi.1009089)
Supplement: S3 Fig — (PDF) [file pcbi.1009089.s008.pdf]

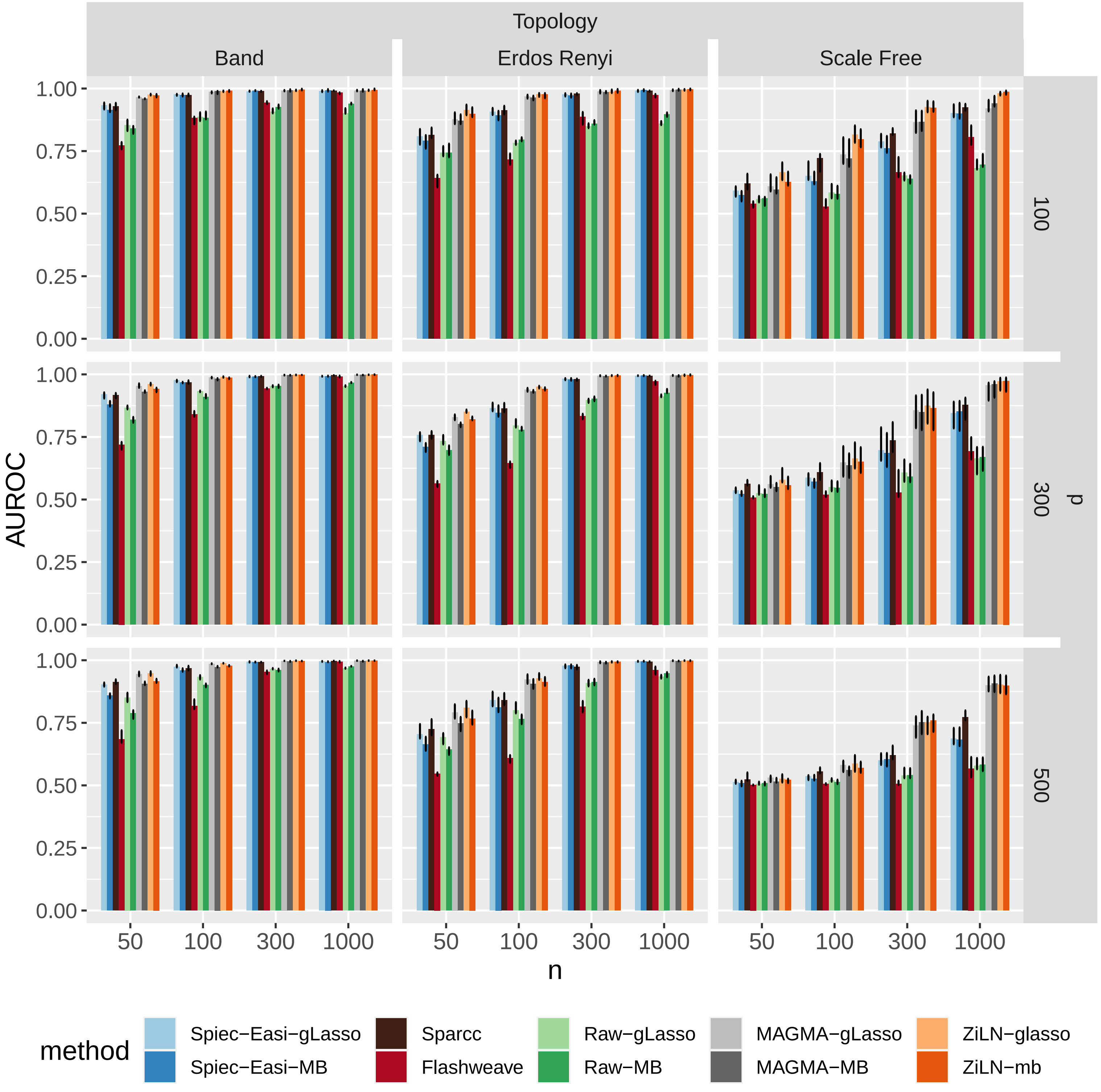

Topology
Band
Erdos Renyi
Scale Free
100
300
500
p
AUROC
1.00
0.75
0.50
0.25
0.00
50
100
300
1000
n
method
Spiec−Easi−gLasso
Spiec−Easi−MB
Sparcc
Flashweave
Raw−gLasso
Raw−MB
MAGMA−gLasso
MAGMA−MB
ZiLN−glasso
ZiLN−mb
