# Supplementary material for: A zero inflated log-normal model for inference of sparse microbial association networks
Source: PLoS Comput Biol. 2021 Jun 18;17(6):e1009089. doi: 10.1371/journal.pcbi.1009089 (PMC8244920; doi:10.1371/journal.pcbi.1009089)
Supplement: Attachment — Submitted filename: prost_answers2reviewers.pdf [file pcbi.1009089.s009.pdf]

# Response to reviewers

Vincent Prost

April 2021

First of all, we would like to thank both reviewers for their valuable and encouraging feedback. We provide hereunder answers to their questions and comments, which have led to substantial editions in the manuscript and additional tables and figures (panel A of Fig1, Appendix E and figures of Appendix F).

## 1 Reviewer 1

#### 1.0.1

The authors tended to use "association" and "ecological interaction" interchangeably. As it is noted in recent works (Carr et al, 2019; Hirano & Takemoto, 2019), associations between microbial abundances do not necessarily mean interactions.

**Answer** We certainly agree with this remark, and have replaced the excessive terms "ecological interaction" with the word "interaction" where appropriate.

#### 1.0.2

Introduction Line 76: How to tell a zero is a structural zero or a sampling zero? In practice, sampling zeros are essentially treated the same as structural zeros by this algorithm? Then how would the sequencing depth affect the performance of the algorithm?

**Answer** In gaussian graphical models, zeros are interpreted as biological zeros and are related to a latent gaussian variable, while the read counts can be modeled with a poisson or multinomial distribution whose parameter is proportional to the abundance proportions of the microbes present in the sample. In this generative model description, the sampling zeros are modelled at the count level. But the reviewer is right that strictly speaking at the inference level all zeros are treated as structural. On the other hand, as both types of zeros technically imply a rather modest latent variable for low abundance taxa, we don't expect big differences between both types of zeroes in that case.

### 1.0.3

Figure 1: as this figure only shows some examples, the authors might consider calculate a distance between simulated and true distributions, and show a comparison of the distances across all taxa.

**Answer** The count distributions shown in Figure 1 indeed do not provide a comprehensive assessment of the differences between real and simulated counts. We therefore measured the feature by feature differences between these distributions with the Kolmogorov-Smirnov statistic (i.e. the largest absolute difference between the observed cdf of the real and simulated taxa abundances), providing a distance between them. The panel A of Figure 1 shows that the proposed method achieves a smaller per-feature difference with the real distribution.

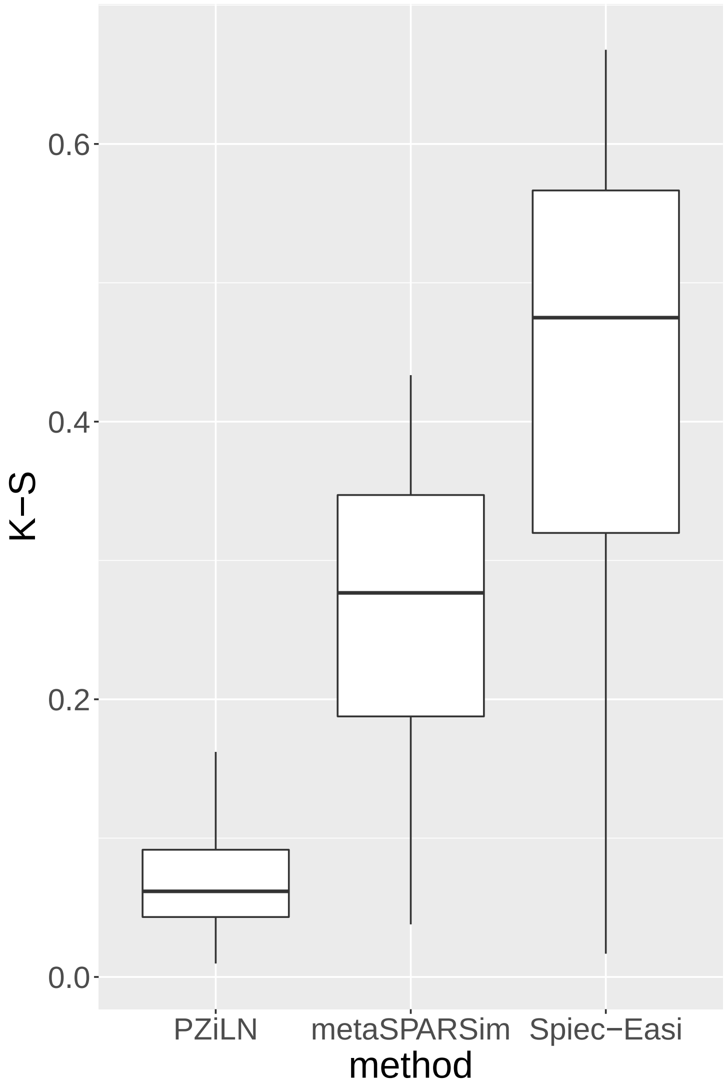


Figure 1: K-S statistic per taxa

**1.0.4**

In synthetic dataset 1 and 2, what was the number of edges in each simulation? Will this (i.e. the network density) affect the performance of the inference?

**Answer** The sparsity assumption of the association graphs translates into a scaling of the number of taxon-taxon associations (edges) with the number of taxa (nodes), i.e. $e = p$. The sparsity of the networks is controlled by

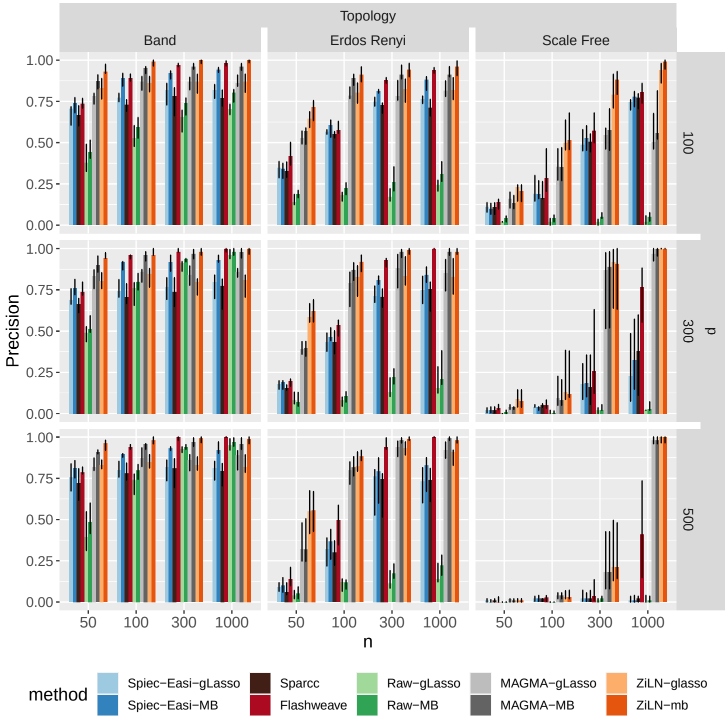


Figure 2: Precision of the various methods on the top 50 edge predictions.

the number of edges, and can affect very significantly the performance of the inference by the various methods as shown in figure 3.

### 1.0.5

While AUPR is good for measuring the performance in some perspectives, it would be great if the authors could consider other metrics. For instance, the precision and recall (and F1 maybe) of the top 50 predictions is probably more important for practical usage.

**Answer** We initially also computed other metrics for evaluating the performance of our method, like AUROC values now shown in Figure 3 of Appendix F. As suggested by the reviewer, we also computed precision for the top 50 edge predictions (shown in Figure 2 of Appendix F). Both of these measures are largely consistent with the AUPR-based results shown in Figure 2 of the main text.

#### 1.0.6

Fig 4: It would be helpful to run some network clustering algorithm or community detection algorithm to demonstrate that phylogenetically related species were more often in the same cluster/community than random.

**Answer** In order to provide a more quantitative support for the observed assortativity phenomenon, we computed assortativity coefficients (graph homophyly) for the different methods at various phylogenetic levels (also shown in Appendix E). These measures indicate statistically significant ($p < 10^{-4}$) assortativity for all the methods.

| Level | Spiec-Easi | Flashweave | MAGMA | ZiLN |
|---|---|---|---|---|
| Phylum | 0.14 | 0.15 | 0.19 | 0.15 |
| Class | 0.09 | 0.11 | 0.13 | 0.12 |
| Order | 0.08 | 0.10 | 0.11 | 0.10 |

Table 1: Assortativity coefficients

### 1.1 Minor concerns

#### 1.1.1

What is the $\delta_j$ in equation 1?

**Answer** $\delta_j$ in equation 1 is the parameter determining the zero probability.

#### 1.1.2

Introduction Line 9: I guess the authors mean "mutualism" (positive-positive interaction) instead of "commensalism" (neutral-positive interaction). Why are these four types of interactions "most important"?

**Answer** Thank you for pointing this possible confusion. In order to improve clarity, we chose to now present the (pairwise) interaction types according to Lidicker's famous wheel ([4]), with mutualism (+/+), competition (-/-), predation or parasitism (+/-) or (-/+), amenalism (0/-) or (-/0) and commensalism (+/0) or (0/+) interaction types. We also presented rather naively these interactions as "most important" and have corrected this in the new version.

#### 1.1.3

Section 3.2.1: The first paragraph seems largely repetitive with the text in the introduction

**Answer** Thank you for spotting this redundancy; we eliminated the culprit paragraph in the introduction (originally from lines 65 to 73).

### 1.1.4

Methods: How was the EM algorithm stopped?

**Answer** We think (hope) that an interesting aspect of the presented work is that the data transformation, initial parameter estimates and posterior mean transformation allow a one-step EM computation (see section 3.2.5 and Appendix C). This follows from Sinclair et al. ([9]) who showed that -from an appropriate starting point- a one-step EM algorithm corresponds to a data transformation provably outperforming the starting point after the glasso is applied [9].

# 2 Reviewer 2

Reviewer #2: This paper presents a truncated log-normal model to evaluate networks for microbiome data that accounts for compositionality and the zero-inflated counts. This model can be used to make inferences about microbial interaction networks and to simulate microbiome data. The authors compare simulated data results and application to a motivating dataset across different approaches. The paper is very well written and the model description is clear. I offer a few suggestions or points that might be helpful to clarify below.

**Answer** Thank you very much for your feedback and encouraging comment!

### 2.0.1

How are the covariances between taxa affects by the 0 counts? Would rare taxa tend to have higher covariances simply because they tend to be absent from the majority of samples rather than due to an actual interaction? In other words, for two rare taxa that do not co-occur in any samples, is their covariance greater than 0?

**Answer** We should point here that, if two rare taxa do not co-occur in any samples, their empirical covariance will be strictly negative:

Given $\boldsymbol{X} = (x_1, x_2, .., x_N)$, and $\boldsymbol{Y} = (y_1, y_2, .., y_N)$ two samples with no co-occurence, $\hat{\mu}_X = \frac{\sum_i x_i}{N}$ and $\hat{\mu}_Y = \frac{\sum_i y_i}{N}$, then

$$\hat{\text{Cov}}(\boldsymbol{X}, \boldsymbol{Y}) = \frac{1}{N-1}\left(\sum_i x_i y_i - \hat{\mu}_X \hat{\mu}_Y\right)$$

We have thus $\sum x_i y_i = 0$, and therefore $\hat{\text{Cov}}(\boldsymbol{X}, \boldsymbol{Y}) = -\frac{N}{N-1}\hat{\mu}_X \hat{\mu}_Y < 0$ because $\hat{\mu}_X, \hat{\mu}_Y > 0$.

### 2.0.2

The authors present the clr transformation to account for the compositionality of the abundance data, there are other transformations (ilr) that are equally common. I was wondering if the authors could comment on whether their approach can similarly be applied to the other transformations or if some of the attributes are specific to using the clr?

**Answer** We acknowledge that there are alternative compositionally robust log-ratio transformations to the CLR in order to analyze changes in relative abundances of taxa. The CLR can have some drawbacks (e.g. singular covariance matrix) and the ILR, which is built from a sequential binary partition of the original input space, can circumvent some of these[7]. Some successful applications of the ILR and/or of balances have been described ([8, 6]). However, difficult interpretation issues are frequently associated to ILR[6], as the meaningfulness of the new coordinate system relies on the choice of the partition system (an orthonormal basis), which is not necessarily trivial or intuitive to define. It is important to note also that ILR has no specific solution to the problem of zeroes and low coverga samples. In several instances, ad-hoc partitioning have lead to difficult to interpret partitions ([3]), or external phylogenetic trees have been used as binary partitions to define neighboring groups of taxa to investigate ([8]). In contrast, beyond the straightforward one-to-one mapping between CLR coordinates and individual taxa, the fact that the latter can be efficiently computed is also an attractive aspect.

An interesting side note in relation to the assortativity issue raised by both reviewers is the fact that the decomposition of variance by [8] indicated that covariation of bacterial clades within human body sites increased with phylogenetic relatedness.

### 2.0.3

I don't quite understand how the real counts in Fig 1 are aligned with the simulated counts, more specifically how are the real counts from taxa 540 matched up with the simulated counts from each approach. Did the authors use some information about the taxa from the real data to simulate counts for the same taxa? Or is there actually no pairing of information within each panel and the figure is meant to represent a range of distributions?

**Answer** The fitting of the simulated data on the sample by taxon matrix corresponding to real data actually yields simulated data matrices having the same dimensionality as the real one, with the columns of the simulated matrices corresponding to the columns (taxa) of the real data matrix, therefore providing a pairing mechanism.

### 2.0.4

In Section 3.3.1, it wasn't clear to me why the size for the negative binomial random number generator equaled 5. Same question for the parameters used to select the mean of the Gaussian distribution. Were these based on estimates from the real dataset?

**Answer** The size parameter for the negative binomial was set to 5 rather arbitrary, as a small enough value was selected in order to have a significant variance component: for the real data, we have $\text{mean}(N_i) = 6.10^6$ and $\text{Var}(N_i) = 5.10^{12}$, so if $X \sim \mathcal{NB}(\text{mean} = \mu, \text{size} = r)$ then the variance of $\text{Var}(X) = \mu(1 + \frac{\mu}{r})$

As for the gaussian, picking $\mu$ in the [0, 3] interval results in a mean abundance of taxa of $e^{\mu+\frac{\sigma^2}{2}}$, capturing both high and low abundance taxa as the mean maximal ratio between low and high abundance taxa is about $e^3 \approx 20$.

### 2.0.5

In the results, the authors refer to methods being compared using the reference numbers (line 265), it would be helpful to also include the name of the method as it appears in the referenced figure so the reader doesn't have to refer to earlier text.

**Answer** Thank you for pointing this, which is corrected now.

### 2.0.6

I don't have as much experience with the network approaches as other parts of the model, so my apologies if this is a well-known concept in the network literature. I don't understand what information is being used to estimate the AUPR, the authors mention it is a measure of accuracy of network structure. Does it focus more on recovering the edges assuming all the nodes are the same or is that true only in this context since the nodes don't change. A little bit more explanation or a reference would be helpful.

**Answer** When dealing with datasets suffering from severe class imbalances (e.g. microbial association networks are dominated by true negatives), precision-recall (PR) curves give a more informative picture of an algorithm's performance, and methods optimizing the area under the ROC curve are not guaranteed to optimize the area under the PR curve [1]. This is provoked by how true negatives are treated, as a large change in the number of false positives can lead to only a small change in the false positive rate used in ROC analysis, while precision (by comparing false positives to true positives rather than true negatives) captures the effect of the large number of negative examples on the performance.

In order to clarify how the AUPR is computed, we tried to improve the description of the accuracy computation and the model selection process underpinning it. The process does not rely on the inference of a single graph but of a series of graphical models of varying sparsity (including the empty and complete network as extremes), which together constitute the solution path. Each of the decreasing values of the sparsity-controlling parameter in the solution path leads to a graph solution, including its set of edges. If we denote the latter by $E_i$ for graph solution $i$, we therefore have $E_1 \subset E_2 \subset E_i.. \subset E_n$, which provides a natural ordering of the set of edges: edges associated to elevated sparsity-controling parameters are more reliable than edges appearing ectopically under low sparsity settings. This provides a rationale for ordering the edges and computing AUPR values. Alternatively, the so-called StARS scheme (Stability Approach to Regularization Selection, [5]) can also be used to repeatedly take a random subset of the data and estimate the solution path. For each repetition, the edge frequencies across the network solution path are measured in order to define an overall edge stability, and the sparsity value at which the graphs from different samplings are the least variable is identified and used for model selection. This way, the edge frequencies of the selected model indicate their reproducibility and can be used to rank the edges for computing accuracy values.

### 2.0.7

It would also be helpful to have some additional information for the real-world example related to the sparsity of the counts, a description of the average number of sequences per sample and the total number of taxa. This would help the reader put this data in context with the simulated data presented earlier in the paper.

**Answer** Thank you for pointig this omission, which we have corrected. The LifeLines-DEEP cohort includes 1,135 individuals (474 men and 661 women) from the general Dutch population, whose gut microbiomes were shotgun sequenced using the Illumina short-read technology, generating an average of 32 million reads per sample ([10], corresponding to EBI dataset accession EGAD00001001991). We phylogenetically profiled the sequences from the LifeLines-Deep cohort using the metapalette software [2], and several samples by taxa matrices corresponding to different taxonomic ranks were constructed from these results, e.g. the raw species-level matrix contains 3957 taxa (variables) and about 90% zero entries.

### 2.0.8

Do the authors have an idea of how the results presented in Figure 5 might change using different values to define edges? It wasn't clear how the presence of an edge was defined and whether this was consistently used for all approaches.

**Answer** First, to be completely transparent on the parameters used for the various methods, we now provide the script used to call them on the repository.

In order to get comparable edge sets for the different methods, we simply decided to pick the 1200 top edges for each method (e.g. this entails using a sparsity-controlling parameter yielding 1200 detected edges). Next, in order to provide a more quantitative support for the results shown in the Venn diagram (figure 5) about the observed assortativity phenomenon, we computed assortativity coefficients (using the igraph R package) for the different methods at various phylogenetic levels. These measures are shown in Appendix E, indicating that assortativity is statistically significant ($p < 10^{-4}$) for all methods.

# References


[1] Jesse Davis and Mark Goadrich. The relationship between precision-recall and roc curves. In *Proceedings of the 23rd international conference on Machine learning*, pages 233–240, 2006.

[2] David Koslicki and Daniel Falush. Metapalette: a k-mer painting approach for metagenomic taxonomic profiling and quantification of novel strain variation. *MSystems*, 1(3), 2016.

[3] Kim-Anh Lê Cao, Mary-Ellen Costello, Vanessa Anne Lakis, Francois Bartolo, Xin-Yi Chua, Remi Brazeilles, and Pascale Rondeau. Mixmc: a multivariate statistical framework to gain insight into microbial communities. *PloS one*, 11(8):e0160169, 2016.

[4] William Z Lidicker Jr. A clarification of interactions in ecological systems. *Bioscience*, 29(8):475–477, 1979.

[5] Han Liu, Kathryn Roeder, and Larry Wasserman. Stability approach to regularization selection (stars) for high dimensional graphical models. *Advances in neural information processing systems*, 24(2):1432, 2010.

[6] James T Morton, Jon Sanders, Robert A Quinn, Daniel McDonald, Antonio Gonzalez, Yoshiki Vázquez-Baeza, Jose A Navas-Molina, Se Jin Song, Jessica L Metcalf, Embriette R Hyde, et al. Balance trees reveal microbial niche differentiation. *MSystems*, 2(1), 2017.

[7] Vera Pawlowsky-Glahn, Juan José Egozcue, and Raimon Tolosana-Delgado. *Modeling and analysis of compositional data.* John Wiley & Sons, 2015.

[8] Justin D Silverman, Alex D Washburne, Sayan Mukherjee, and Lawrence A David. A phylogenetic transform enhances analysis of compositional microbiota data. *Elife*, 6:e21887, 2017.

[9] David Sinclair and Giles Hooker. Sparse inverse covariance estimation for high-throughput microrna sequencing data in the poisson log-normal graphical model. *Journal of Statistical Computation and Simulation*, 89(16):3105–3117, 2019.

[10] Alexandra Zhernakova, Alexander Kurilshikov, Marc Jan Bonder, Ettje F Tigchelaar, Melanie Schirmer, Tommi Vatanen, Zlatan Mujagic, Arnau Vich Vila, Gwen Falony, Sara Vieira-Silva, et al. Population-based metagenomics analysis reveals markers for gut microbiome composition and diversity. *Science*, 352(6285):565–569, 2016.
